# Poly[[diaqua­(μ_2_-5,5-dioxodibenzo[*b*,*d*]thio­phene-3,7-dicarboxyl­ato)(μ_2_-ethyl­ene glycol)manganese(II)] dimethyl­acetamide solvate]

**DOI:** 10.1107/S1600536810045411

**Published:** 2010-11-13

**Authors:** Xiu-Chun Yi, Li Yan, En-Qing Gao

**Affiliations:** a Shanghai Key Laboratory of Green Chemistry and Chemical Processes, Department of Chemistry, East China Normal University, Shanghai 200062, People’s Republic of China

## Abstract

In the title complex, {[Mn(C_14_H_6_O_6_S)(C_2_H_6_O_2_)(H_2_O)_2_]·C_4_H_9_NO}_*n*_, the Mn^II^ ion is six-coordinated in a *trans*-octa­hedral geometry by two carboxyl­ate O atoms from two 5,5-dioxodibenzo[*b*,*d*]thio­phene-3,7-dicarboxyl­ate (*L*) ligands in a monodentate mode, two O atoms from two ethyl­ene glycol (EG) mol­ecules and two aqua O atoms. The metal ions are linked by the EG and *L* ligands, forming two-dimensional coordination networks, which are associated into the three-dimensional structure through O—H⋯O hydrogen bonds.

## Related literature

For the use of H_2_ *L* ligands in the construction of coordination polymers, including metal-organic frameworks with function­alized pores, see: Neofotistou *et al.* (2010 ▶). Kanaizuka *et al.* (2010 ▶). Yan *et al.* (2009 ▶). For the ligand synthesis, see: Neofotistou *et al.* (2009 ▶). 
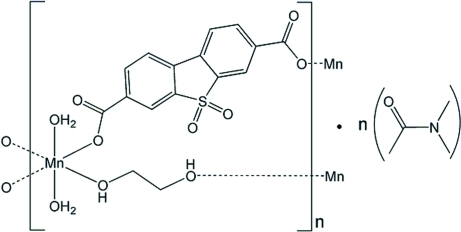


## Experimental

### 

#### Crystal data

[Mn(C_14_H_6_O_6_S)(C_2_H_6_O_2_)(H_2_O)_2_]·C_4_H_9_NO
*M* *_r_* = 542.41
Monoclinic, 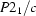

*a* = 7.0564 (3) Å
*b* = 11.8142 (4) Å
*c* = 28.0008 (10) Å
β = 100.544 (1)°
*V* = 2294.89 (15) Å^3^
*Z* = 4
Mo *K*α radiation
μ = 0.73 mm^−1^
*T* = 296 K
0.30 × 0.20 × 0.10 mm

#### Data collection

Bruker APEXII CCD area-detector diffractometer
Absorption correction: multi-scan (*SADABS*; Bruker, 2008 ▶) *T*_min_ = 0.811, *T*_max_ = 0.931
30673 measured reflections
5716 independent reflections
5221 reflections with *I* > 2σ(*I*)
*R*_int_ = 0.025

#### Refinement

*R*[*F* ^2^ > 2σ(*F* ^2^)] = 0.030
*wR*(*F* ^2^) = 0.083
*S* = 1.07
5716 reflections
335 parameters
4 restraints
H atoms treated by a mixture of independent and constrained refinement
Δρ_max_ = 0.32 e Å^−3^
Δρ_min_ = −0.36 e Å^−3^

### 

Data collection: *APEX2* (Bruker, 2007 ▶); cell refinement: *SAINT* (Bruker, 2007 ▶); data reduction: *SAINT*; program(s) used to solve structure: *SHELXS97* (Sheldrick, 2008 ▶); program(s) used to refine structure: *SHELXL97* (Sheldrick, 2008 ▶); molecular graphics: *SHELXTL* (Sheldrick, 2008 ▶); software used to prepare material for publication: *SHELXTL*.

## Supplementary Material

Crystal structure: contains datablocks I, global. DOI: 10.1107/S1600536810045411/fk2028sup1.cif

Structure factors: contains datablocks I. DOI: 10.1107/S1600536810045411/fk2028Isup2.hkl

Additional supplementary materials:  crystallographic information; 3D view; checkCIF report

## Figures and Tables

**Table 1:** Selected bond lengths (Å)

Mn1—O9	2.1191 (12)
Mn1—O3^i^	2.1437 (10)
Mn1—O1	2.1710 (10)
Mn1—O10	2.1897 (12)
Mn1—O8^ii^	2.2138 (12)
Mn1—O7	2.2253 (11)

Symmetry codes: (i) 
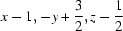
; (ii) 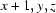.

**Table 2:** Hydrogen-bond geometry (Å, °)

*D*—H⋯*A*	*D*—H	H⋯*A*	*D*⋯*A*	*D*—H⋯*A*
O7—H7⋯O4^iii^	0.84 (2)	1.83 (2)	2.6623 (16)	175 (2)
O8—H8⋯O2^iv^	0.82 (2)	1.88 (2)	2.6865 (16)	169 (2)
O9—H9*C*⋯O11	0.83 (2)	1.89 (2)	2.7086 (17)	171 (2)
O9—H9*B*⋯O2	0.83 (2)	1.84 (2)	2.6135 (16)	156 (2)
O10—H10*C*⋯O11^iv^	0.83 (3)	1.96 (3)	2.7877 (19)	172 (2)
O10—H10*B*⋯O4^i^	0.82 (3)	1.98 (3)	2.7663 (16)	161 (3)

Symmetry codes: (i) 
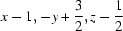
; (iii) 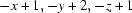; (iv) 
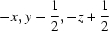
.

**Fig. 1.**
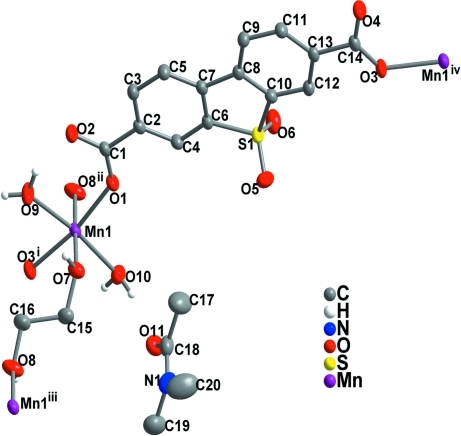
Coordination enviroment in the title compound showing the atom-labelling scheme. Displacement ellipsoids are drawn at the 50% probability level and the H atoms attached to C are omitted for clarity. [Symmetry code: (i)-1/2 + x, 1/2 - y, 1/2 + z (ii) 1 + x, y, z (iii) -1 + x, y, z (iv)1/2 + x, 1/2 - y, -1/2 + z]

**Fig. 2.**
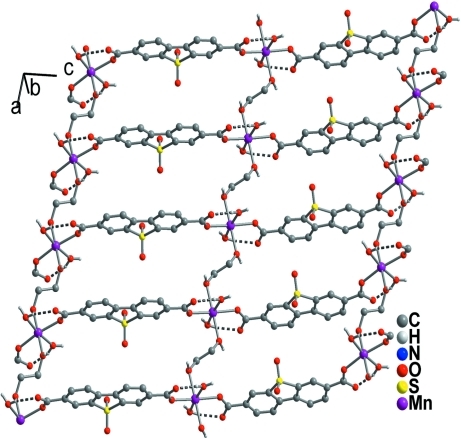
Two-dimensional layer connected through L and EG. The intralayer hydrogen bonds are shown as dot lines. The H atoms attached to C are omitted for clarity.

## supplementary crystallographic information

### Comment

In this paper, we report the coordination and hydrogen-bond structure of the title complex (I) derived from *S*,*S*-dioxodibenzothiophen-3,7-dicarboxylic acid (H_2_L). These sulfone-functionalized dicarboxylic ligands have recently been used to construct coordination polymers, including metal-organic frameworks with functionalized pores (Kanaizuka *et al.* (2010); Neofotistou *et al.* (2010); Neofotistou *et al.* (2009); Yan *et al.* (2009)). The asymmetric unit of I contains a Mn(II) ion, an *L* ligand, two aqua ligands, an ethylene glycol (EG) ligand, and an *N*,*N*-dimethylacetamide (DMA) solvent molecule (Fig. 1). Each Mn atom resides in a *trans*-octahedral coordination geometry completed by two carboxylate O atoms (O1 and O3) from two *L* ligands, two O atoms (O7 and O8) from two EG molecules, and two O atoms (O9 and O10) from two water molecules. The Mn—O distances lie in the range of 2.1191 (12)–2.2253 (11) Å (Table 1). The *L* ligand binds two Mn atoms through two monodentate carboxylate groups, and the EG ligand also binds two Mn atoms through its two hydroxyl groups. Consequently, the metal ions are linked into a two-dimensional layer (Fig. 2). The carboxylate, hydroxyl, and aqua groups from the coordination sphere and the carbonyl group from the DMA molecule provide plenty of sites for hydrogen bonding (Table 2). Each uncoordinated carboxylate oxygen atom (O2 or O4) serves as a bifurcate acceptor to form an intralayer hydrogen bond with a coordinated aqua molecule and an interlayer one with a EG hydroxyl group from the neighboring layer. The oxygen atom (O11) of the DMA solvent is also bifurcately hydrogen-bonded, to two independent aqua ligands from different coordination layers. The above hydrogen bonds collaborate to assemble the two-dimensional coordination layers into a three-dimensional structure.

### Experimental

The ligand was synthesized from dimethyl ester of 4,4'-biphenyldicarboxylic acid and H_2_SO_4, _20%SO_3_ (oleum) according to the procedure for similar compounds (Neofotistou *et al.*, 2009). The ligand (0.006 g, 0.03 mmol) and MnCl_2_.6H_2_O (0.006 g, 0.03 mmol) were dissolved under stirring in a mixture of ethylene glycol (1.0 ml) and DMA (2.0 ml). The resulting solution was left to stand at room temperature for 20 days to afford colorless block crystals of the title compound.

### Refinement

All hydrogen atoms attached to carbon atoms were placed at calculated positions and refined with the riding model using AFIX 43 and AFIX 23 instructions for aromatic C—H and secondary CH_2_. The hydrogen atoms from EG and water were initially located from difference Fourier maps and refined isotropically with restraints on O—H distance (0.85 Å) and H—O—H angles.

### Crystal data

**Table:**

[Mn(C_14_H_6_O_6_S)(C_2_H_6_O_2_)(H_2_O)_2_]·C_4_H_9_NO	*F*(000) = 1124
*M**_r_* = 542.41	*D*_x_ = 1.570 Mg m^−^^3^
Monoclinic, *P*2_1_/*c*	Mo *K*α radiation, λ = 0.71073 Å
Hall symbol: -P 2ybc	Cell parameters from 9208 reflections
*a* = 7.0564 (3) Å	θ = 2.3–26°
*b* = 11.8142 (4) Å	µ = 0.73 mm^−^^1^
*c* = 28.0008 (10) Å	*T* = 296 K
β = 100.544 (1)°	Columnar, colourless
*V* = 2294.89 (15) Å^3^	0.30 × 0.20 × 0.10 mm
*Z* = 4	

### Data collection

**Table:**

Bruker APEXII CCD area-detector diffractometer	5716 independent reflections
Radiation source: fine-focus sealed tube	5221 reflections with *I* > 2σ(*I*)
graphite	*R*_int_ = 0.025
φ and ω scans	θ_max_ = 28.4°, θ_min_ = 1.5°
Absorption correction: multi-scan (*SADABS*; Bruker, 2008)	*h* = −9→7
*T*_min_ = 0.811, *T*_max_ = 0.931	*k* = −15→14
30673 measured reflections	*l* = −37→36

### Refinement

**Table:**

Refinement on *F*^2^	Secondary atom site location: difference Fourier map
Least-squares matrix: full	Hydrogen site location: geom and difmap
*R*[*F*^2^ > 2σ(*F*^2^)] = 0.030	H atoms treated by a mixture of independent and constrained refinement
*wR*(*F*^2^) = 0.083	*w* = 1/[σ^2^(*F*_o_^2^) + (0.0425*P*)^2^ + 1.0021*P*] where *P* = (*F*_o_^2^ + 2*F*_c_^2^)/3
*S* = 1.07	(Δ/σ)_max_ = 0.001
5716 reflections	Δρ_max_ = 0.32 e Å^−^^3^
335 parameters	Δρ_min_ = −0.36 e Å^−^^3^
4 restraints	Extinction correction: *SHELXL97* (Sheldrick, 2008), Fc^*^=kFc[1+0.001xFc^2^λ^3^/sin(2θ)]^-1/4^
Primary atom site location: structure-invariant direct methods	Extinction coefficient: 0.0011 (3)

### Special details

**Table:**

Geometry. All e.s.d.'s (except the e.s.d. in the dihedral angle between two l.s. planes) are estimated using the full covariance matrix. The cell e.s.d.'s are taken into account individually in the estimation of e.s.d.'s in distances, angles and torsion angles; correlations between e.s.d.'s in cell parameters are only used when they are defined by crystal symmetry. An approximate (isotropic) treatment of cell e.s.d.'s is used for estimating e.s.d.'s involving l.s. planes.
Refinement. Refinement of *F*^2^ against ALL reflections. The weighted *R*-factor *wR* and goodness of fit *S* are based on *F*^2^, conventional *R*-factors *R* are based on *F*, with *F* set to zero for negative *F*^2^. The threshold expression of *F*^2^ > σ(*F*^2^) is used only for calculating *R*-factors(gt) *etc*. and is not relevant to the choice of reflections for refinement. *R*-factors based on *F*^2^ are statistically about twice as large as those based on *F*, and *R*- factors based on ALL data will be even larger.

### Fractional atomic coordinates and isotropic or equivalent isotropic displacement parameters (Å^2^)

**Table:**

	*x*	*y*	*z*	*U*_iso_*/*U*_eq_	
Mn1	0.22688 (3)	0.673463 (17)	0.267191 (7)	0.02227 (7)	
C1	0.43784 (19)	0.86846 (12)	0.33374 (5)	0.0236 (3)	
C2	0.53981 (19)	0.90349 (12)	0.38359 (5)	0.0225 (3)	
C3	0.5765 (2)	1.01741 (12)	0.39461 (5)	0.0249 (3)	
H3A	0.5433	1.0713	0.3703	0.030*	
C4	0.5922 (2)	0.82195 (11)	0.41959 (5)	0.0247 (3)	
H4A	0.5704	0.7454	0.4130	0.030*	
C5	0.6613 (2)	1.05261 (12)	0.44092 (5)	0.0247 (3)	
H5A	0.6842	1.1290	0.4476	0.030*	
C6	0.67736 (19)	0.85829 (12)	0.46533 (5)	0.0229 (3)	
C7	0.71153 (19)	0.97220 (11)	0.47712 (5)	0.0216 (2)	
C8	0.79778 (19)	0.98999 (11)	0.52870 (5)	0.0213 (2)	
C9	0.8502 (2)	1.09104 (11)	0.55269 (5)	0.0244 (3)	
H9A	0.8314	1.1596	0.5362	0.029*	
C10	0.8262 (2)	0.88924 (11)	0.55495 (5)	0.0229 (3)	
C11	0.9316 (2)	1.08823 (12)	0.60187 (5)	0.0242 (3)	
H11A	0.9687	1.1557	0.6180	0.029*	
C12	0.90422 (19)	0.88466 (12)	0.60378 (5)	0.0235 (3)	
H12A	0.9199	0.8161	0.6203	0.028*	
C13	0.95877 (18)	0.98663 (11)	0.62747 (5)	0.0215 (3)	
C14	1.05227 (19)	0.98394 (12)	0.68052 (5)	0.0224 (3)	
O1	0.39522 (15)	0.76549 (9)	0.32753 (4)	0.0290 (2)	
O2	0.39864 (18)	0.94438 (10)	0.30224 (4)	0.0366 (3)	
O3	1.05942 (16)	0.88879 (9)	0.70073 (4)	0.0316 (2)	
O4	1.11941 (17)	1.07339 (9)	0.70058 (4)	0.0339 (2)	
O5	0.5980 (2)	0.71159 (11)	0.53019 (4)	0.0437 (3)	
O6	0.92266 (19)	0.70701 (10)	0.51083 (4)	0.0411 (3)	
S1	0.75616 (5)	0.77206 (3)	0.516692 (12)	0.02602 (9)	
C15	−0.2116 (2)	0.65002 (14)	0.28938 (6)	0.0334 (3)	
H15A	−0.2780	0.6571	0.3167	0.040*	
H15B	−0.1888	0.5702	0.2847	0.040*	
C16	−0.3383 (2)	0.69648 (15)	0.24442 (6)	0.0334 (3)	
H16A	−0.3629	0.7761	0.2490	0.040*	
H16B	−0.2730	0.6894	0.2169	0.040*	
O7	−0.03049 (15)	0.70750 (10)	0.30054 (4)	0.0312 (2)	
H7	−0.052 (3)	0.777 (2)	0.3005 (8)	0.048 (6)*	
O8	−0.51664 (16)	0.63629 (11)	0.23467 (5)	0.0381 (3)	
H8	−0.495 (3)	0.5748 (15)	0.2233 (8)	0.052 (6)*	
O9	0.1821 (2)	0.83273 (10)	0.23213 (5)	0.0471 (3)	
H9C	0.135 (3)	0.854 (2)	0.2043 (6)	0.056 (7)*	
H9B	0.246 (3)	0.8828 (18)	0.2484 (7)	0.057 (7)*	
O10	0.24423 (19)	0.49977 (10)	0.29494 (5)	0.0352 (3)	
H10C	0.182 (3)	0.474 (2)	0.3150 (9)	0.057 (7)*	
H10B	0.210 (4)	0.463 (2)	0.2701 (10)	0.072 (9)*	
C17	−0.0751 (4)	1.0498 (2)	0.08416 (8)	0.0639 (6)	
H17A	−0.1888	1.0591	0.0981	0.096*	
H17B	−0.1115	1.0281	0.0507	0.096*	
H17C	−0.0053	1.1199	0.0863	0.096*	
C18	0.0504 (3)	0.95932 (15)	0.11144 (6)	0.0391 (4)	
C19	0.3395 (3)	0.8464 (2)	0.12584 (8)	0.0578 (5)	
H19A	0.2600	0.7865	0.1344	0.087*	
H19B	0.4142	0.8782	0.1548	0.087*	
H19C	0.4244	0.8168	0.1057	0.087*	
C20	0.2899 (5)	0.9857 (2)	0.05873 (11)	0.0830 (9)	
H20A	0.2025	1.0437	0.0443	0.125*	
H20B	0.3005	0.9288	0.0349	0.125*	
H20C	0.4144	1.0184	0.0703	0.125*	
O11	−0.0072 (2)	0.90934 (12)	0.14522 (4)	0.0461 (3)	
N1	0.2175 (2)	0.93427 (13)	0.09933 (6)	0.0440 (3)	

### Atomic displacement parameters (Å^2^)

**Table:**

	*U*^11^	*U*^22^	*U*^33^	*U*^12^	*U*^13^	*U*^23^
Mn1	0.02329 (11)	0.02213 (11)	0.01900 (11)	−0.00047 (7)	−0.00245 (8)	−0.00162 (7)
C1	0.0221 (6)	0.0278 (7)	0.0191 (6)	−0.0016 (5)	−0.0007 (5)	−0.0006 (5)
C2	0.0211 (6)	0.0267 (6)	0.0184 (6)	−0.0021 (5)	0.0002 (5)	−0.0018 (5)
C3	0.0283 (7)	0.0253 (7)	0.0193 (6)	−0.0009 (5)	−0.0004 (5)	0.0015 (5)
C4	0.0268 (7)	0.0227 (6)	0.0228 (6)	−0.0024 (5)	−0.0006 (5)	−0.0026 (5)
C5	0.0302 (7)	0.0217 (6)	0.0207 (6)	−0.0010 (5)	0.0009 (5)	−0.0008 (5)
C6	0.0244 (6)	0.0235 (6)	0.0190 (6)	0.0005 (5)	−0.0006 (5)	0.0014 (5)
C7	0.0212 (6)	0.0247 (6)	0.0177 (6)	−0.0004 (5)	0.0005 (5)	−0.0012 (5)
C8	0.0216 (6)	0.0236 (6)	0.0178 (6)	0.0000 (5)	0.0013 (5)	−0.0002 (5)
C9	0.0301 (7)	0.0212 (6)	0.0204 (6)	0.0001 (5)	0.0010 (5)	0.0014 (5)
C10	0.0255 (6)	0.0211 (6)	0.0206 (6)	−0.0014 (5)	0.0006 (5)	−0.0020 (5)
C11	0.0276 (6)	0.0225 (6)	0.0209 (6)	−0.0011 (5)	0.0004 (5)	−0.0026 (5)
C12	0.0257 (6)	0.0231 (6)	0.0203 (6)	0.0011 (5)	0.0003 (5)	0.0020 (5)
C13	0.0203 (6)	0.0255 (6)	0.0176 (6)	0.0015 (5)	0.0002 (5)	−0.0005 (5)
C14	0.0213 (6)	0.0256 (6)	0.0187 (6)	0.0031 (5)	−0.0004 (5)	−0.0007 (5)
O1	0.0348 (5)	0.0260 (5)	0.0221 (5)	−0.0033 (4)	−0.0054 (4)	−0.0028 (4)
O2	0.0494 (7)	0.0311 (6)	0.0234 (5)	−0.0091 (5)	−0.0092 (5)	0.0045 (4)
O3	0.0397 (6)	0.0270 (5)	0.0222 (5)	−0.0004 (4)	−0.0094 (4)	0.0035 (4)
O4	0.0472 (6)	0.0264 (5)	0.0234 (5)	−0.0010 (5)	−0.0062 (4)	−0.0033 (4)
O5	0.0572 (8)	0.0372 (6)	0.0347 (6)	−0.0207 (6)	0.0036 (5)	0.0026 (5)
O6	0.0543 (7)	0.0313 (6)	0.0340 (6)	0.0154 (5)	−0.0022 (5)	−0.0037 (5)
S1	0.03603 (19)	0.01997 (16)	0.01953 (16)	−0.00206 (13)	−0.00157 (13)	−0.00020 (11)
C15	0.0273 (7)	0.0319 (7)	0.0406 (8)	−0.0013 (6)	0.0048 (6)	0.0038 (6)
C16	0.0256 (7)	0.0369 (8)	0.0372 (8)	−0.0023 (6)	0.0044 (6)	−0.0012 (6)
O7	0.0269 (5)	0.0278 (5)	0.0375 (6)	0.0006 (4)	0.0022 (4)	−0.0058 (4)
O8	0.0257 (5)	0.0373 (6)	0.0516 (7)	−0.0023 (5)	0.0076 (5)	−0.0186 (5)
O9	0.0779 (10)	0.0270 (6)	0.0266 (6)	−0.0056 (6)	−0.0164 (6)	0.0035 (5)
O10	0.0474 (7)	0.0284 (6)	0.0280 (6)	−0.0012 (5)	0.0018 (5)	0.0029 (5)
C17	0.0792 (16)	0.0631 (14)	0.0481 (12)	0.0237 (12)	0.0083 (11)	0.0178 (10)
C18	0.0535 (10)	0.0349 (8)	0.0253 (7)	0.0019 (7)	−0.0021 (7)	−0.0032 (6)
C19	0.0607 (13)	0.0621 (13)	0.0526 (12)	0.0184 (11)	0.0155 (10)	0.0050 (10)
C20	0.103 (2)	0.0651 (16)	0.095 (2)	0.0174 (15)	0.0574 (18)	0.0312 (15)
O11	0.0525 (8)	0.0550 (8)	0.0298 (6)	0.0113 (6)	0.0049 (5)	0.0074 (5)
N1	0.0569 (10)	0.0382 (8)	0.0377 (8)	0.0020 (7)	0.0109 (7)	−0.0011 (6)

### Geometric parameters (Å, °)

**Table:**

Mn1—O9	2.1191 (12)	C14—O3	1.2553 (17)
Mn1—O3^i^	2.1437 (10)	O3—Mn1^iii^	2.1437 (10)
Mn1—O1	2.1710 (10)	O5—S1	1.4329 (13)
Mn1—O10	2.1897 (12)	O6—S1	1.4382 (13)
Mn1—O8^ii^	2.2138 (12)	C15—O7	1.4303 (18)
Mn1—O7	2.2253 (11)	C15—C16	1.508 (2)
C1—O2	1.2529 (17)	C15—H15A	0.9700
C1—O1	1.2576 (18)	C15—H15B	0.9700
C1—C2	1.5066 (18)	C16—O8	1.4275 (19)
C2—C3	1.3943 (19)	C16—H16A	0.9700
C2—C4	1.3945 (19)	C16—H16B	0.9700
C3—C5	1.3889 (19)	O7—H7	0.84 (2)
C3—H3A	0.9300	O8—Mn1^iv^	2.2138 (11)
C4—C6	1.3799 (18)	O8—H8	0.818 (16)
C4—H4A	0.9300	O9—H9C	0.827 (15)
C5—C7	1.3871 (18)	O9—H9B	0.827 (15)
C5—H5A	0.9300	O10—H10C	0.83 (3)
C6—C7	1.3961 (19)	O10—H10B	0.82 (3)
C6—S1	1.7671 (14)	C17—C18	1.504 (3)
C7—C8	1.4762 (17)	C17—H17A	0.9600
C8—C9	1.3864 (18)	C17—H17B	0.9600
C8—C10	1.3941 (18)	C17—H17C	0.9600
C9—C11	1.3919 (18)	C18—O11	1.245 (2)
C9—H9A	0.9300	C18—N1	1.318 (2)
C10—C12	1.3784 (18)	C19—N1	1.461 (3)
C10—S1	1.7648 (13)	C19—H19A	0.9600
C11—C13	1.3932 (19)	C19—H19B	0.9600
C11—H11A	0.9300	C19—H19C	0.9600
C12—C13	1.3950 (19)	C20—N1	1.462 (3)
C12—H12A	0.9300	C20—H20A	0.9600
C13—C14	1.5114 (17)	C20—H20B	0.9600
C14—O4	1.2488 (17)	C20—H20C	0.9600
			
O9—Mn1—O3^i^	83.73 (4)	C1—O1—Mn1	132.45 (9)
O9—Mn1—O1	85.98 (4)	C14—O3—Mn1^iii^	131.98 (9)
O3^i^—Mn1—O1	169.61 (4)	O5—S1—O6	117.10 (8)
O9—Mn1—O10	172.07 (5)	O5—S1—C10	112.10 (7)
O3^i^—Mn1—O10	88.43 (4)	O6—S1—C10	110.15 (7)
O1—Mn1—O10	101.89 (4)	O5—S1—C6	110.96 (7)
O9—Mn1—O8^ii^	92.82 (6)	O6—S1—C6	110.93 (7)
O3^i^—Mn1—O8^ii^	86.40 (4)	C10—S1—C6	93.06 (6)
O1—Mn1—O8^ii^	92.72 (4)	O7—C15—C16	112.28 (13)
O10—Mn1—O8^ii^	87.89 (5)	O7—C15—H15A	109.1
O9—Mn1—O7	88.29 (6)	C16—C15—H15A	109.1
O3^i^—Mn1—O7	93.65 (4)	O7—C15—H15B	109.1
O1—Mn1—O7	87.44 (4)	C16—C15—H15B	109.1
O10—Mn1—O7	91.00 (5)	H15A—C15—H15B	107.9
O8^ii^—Mn1—O7	178.89 (5)	O8—C16—C15	110.22 (14)
O2—C1—O1	125.37 (12)	O8—C16—H16A	109.6
O2—C1—C2	117.52 (12)	C15—C16—H16A	109.6
O1—C1—C2	117.09 (12)	O8—C16—H16B	109.6
C3—C2—C4	119.54 (12)	C15—C16—H16B	109.6
C3—C2—C1	120.53 (12)	H16A—C16—H16B	108.1
C4—C2—C1	119.90 (12)	C15—O7—Mn1	125.99 (10)
C5—C3—C2	121.77 (13)	C15—O7—H7	108.4 (15)
C5—C3—H3A	119.1	Mn1—O7—H7	109.7 (16)
C2—C3—H3A	119.1	C16—O8—Mn1^iv^	125.51 (10)
C6—C4—C2	117.96 (12)	C16—O8—H8	107.4 (16)
C6—C4—H4A	121.0	Mn1^iv^—O8—H8	123.9 (16)
C2—C4—H4A	121.0	Mn1—O9—H9C	134.6 (16)
C7—C5—C3	119.08 (13)	Mn1—O9—H9B	111.1 (16)
C7—C5—H5A	120.5	H9C—O9—H9B	113 (2)
C3—C5—H5A	120.5	Mn1—O10—H10C	125.2 (17)
C4—C6—C7	123.13 (12)	Mn1—O10—H10B	102 (2)
C4—C6—S1	126.50 (11)	H10C—O10—H10B	106 (2)
C7—C6—S1	110.38 (10)	C18—C17—H17A	109.5
C5—C7—C6	118.52 (12)	C18—C17—H17B	109.5
C5—C7—C8	128.45 (12)	H17A—C17—H17B	109.5
C6—C7—C8	113.03 (12)	C18—C17—H17C	109.5
C9—C8—C10	118.68 (12)	H17A—C17—H17C	109.5
C9—C8—C7	128.49 (12)	H17B—C17—H17C	109.5
C10—C8—C7	112.83 (12)	O11—C18—N1	121.38 (16)
C8—C9—C11	118.95 (12)	O11—C18—C17	118.61 (18)
C8—C9—H9A	120.5	N1—C18—C17	120.01 (18)
C11—C9—H9A	120.5	N1—C19—H19A	109.5
C12—C10—C8	123.32 (12)	N1—C19—H19B	109.5
C12—C10—S1	126.02 (11)	H19A—C19—H19B	109.5
C8—C10—S1	110.64 (10)	N1—C19—H19C	109.5
C9—C11—C13	121.50 (12)	H19A—C19—H19C	109.5
C9—C11—H11A	119.2	H19B—C19—H19C	109.5
C13—C11—H11A	119.2	N1—C20—H20A	109.5
C10—C12—C13	117.59 (12)	N1—C20—H20B	109.5
C10—C12—H12A	121.2	H20A—C20—H20B	109.5
C13—C12—H12A	121.2	N1—C20—H20C	109.5
C11—C13—C12	119.96 (12)	H20A—C20—H20C	109.5
C11—C13—C14	121.20 (12)	H20B—C20—H20C	109.5
C12—C13—C14	118.82 (12)	C18—N1—C19	120.01 (16)
O4—C14—O3	125.06 (12)	C18—N1—C20	124.28 (18)
O4—C14—C13	119.06 (12)	C19—N1—C20	115.66 (19)
O3—C14—C13	115.87 (12)		

Symmetry codes: (i) *x*−1, −*y*+3/2, *z*−1/2; (ii) *x*+1, *y*, *z*; (iii) *x*+1, −*y*+3/2, *z*+1/2; (iv) *x*−1, *y*, *z*.

### Hydrogen-bond geometry (Å, °)

**Table:**

*D*—H···*A*	*D*—H	H···*A*	*D*···*A*	*D*—H···*A*
O7—H7···O4^v^	0.84 (2)	1.83 (2)	2.6623 (16)	175 (2)
O8—H8···O2^vi^	0.82 (2)	1.88 (2)	2.6865 (16)	169 (2)
O9—H9C···O11	0.83 (2)	1.89 (2)	2.7086 (17)	171 (2)
O9—H9B···O2	0.83 (2)	1.84 (2)	2.6135 (16)	156 (2)
O10—H10C···O11^vi^	0.83 (3)	1.96 (3)	2.7877 (19)	172 (2)
O10—H10B···O4^i^	0.82 (3)	1.98 (3)	2.7663 (16)	161 (3)

Symmetry codes: (v) −*x*+1, −*y*+2, −*z*+1; (vi) −*x*, *y*−1/2, −*z*+1/2; (i) *x*−1, −*y*+3/2, *z*−1/2.
